# In-Depth Proteomic Characterization of Classical and Non-Classical Monocyte Subsets

**DOI:** 10.3390/proteomes6010008

**Published:** 2018-02-05

**Authors:** Víctor Segura, M. Luz Valero, Laura Cantero, Javier Muñoz, Eduardo Zarzuela, Fernando García, Kerman Aloria, Javier Beaskoetxea, Jesús M. Arizmendi, Rosana Navajas, Alberto Paradela, Paula Díez, Rosa Mª Dégano, Manuel Fuentes, Alberto Orfao, Andrés García Montero, Alba Garin-Muga, Fernando J. Corrales, Manuel M. Sánchez del Pino

**Affiliations:** 1 Proteomics, Genomics and Bioinformatics Unit, Center for Applied Medical Research, University of Navarra, Pamplona 31008, Spain; vsegura@unav.es (V.S.); agarin@unav.es (A.G.-M.); 2 Proteomics Unit; Central Service for Experimental Research (SCSIE), University of Valencia. Dr Moliner 50, 46100 Burjassot, Spain; mluz.valero@uv.es (M.L.V.); laura.cantero@uv.es (L.C.); 3 Spanish National Cancer Research Centre (CNIO), Melchor Férnandez Almagro, 3, 28029 Madrid. Spain; jmunozpe@cnio.es (J.M.); ezarzuela@cnio.es (E.Z.); fgarcia@cnio.es (F.G.); 4 Proteomics Core Facility-SGIKER, University of the Basque Country, UPV/EHU, 48940 Leioa, Spain; kerman.aloria@ehu.eus (K.A.); 5 Department of Biochemistry and Molecular Biology, University of the Basque Country, UPV/EHU, 48940 Leioa, Spain; javier.beaskoetxea@ehu.eus (J.B.); jm.arizmendi@ehu.eus (J.M.A.); 6 Proteomics Unit, Centro Nacional de Biotecnología-CSIC, Darwin 3, 28049 Madrid, Spain; rnavajas@cnb.csic.es (R.N.); alberto.paradela@cnb.csic.es (A.P.); fcorrales@cnb.csic.es (F.J.C.); 7 Department of Medicine and General Cytometry Service-Nucleus, Cancer Research Centre (IBMCC/CSIC/USAL/IBSAL), 37007 Salamanca, Spain; pauladg@usal.es (P.D.); romade@usal.es (R.Mª.D.); mfuentes@usal.es (M.F.); 8 Proteomics Unit. Cancer Research Centre (IBMCC/CSIC/USAL/IBSAL), 37007 Salamanca, Spain; 9 Cancer Research Center. University of Salamanca-CSIC, IBSAL, 37007 Salamanca, Spain; orfao@usal.es (A.O.); 10 Spanish National DNA Bank Carlos III, University of Salamanca, 37007 Salamanca, Spain; angarmon@usal.es (A.G.M.); 11 Department of Biochemistry and Molecular Biology, University of Valencia. Dr Moliner 50, 46100 Burjassot, Spain; 12 Biotechnology and Biomedicine Interdisciplinary Research Unit (ERI BIOTECMED), University of Valencia. Dr Moliner 50, 46100 Burjassot, Spain

**Keywords:** monocytes, protein profiling, quantitative proteomics

## Abstract

Monocytes are bone marrow-derived leukocytes that are part of the innate immune system. Monocytes are divided into three subsets: classical, intermediate and non-classical, which can be differentiated by their expression of some surface antigens, mainly CD14 and CD16. These cells are key players in the inflammation process underlying the mechanism of many diseases. Thus, the molecular characterization of these cells may provide very useful information for understanding their biology in health and disease. We performed a multicentric proteomic study with pure classical and non-classical populations derived from 12 healthy donors. The robust workflow used provided reproducible results among the five participating laboratories. Over 5000 proteins were identified, and about half of them were quantified using a spectral counting approach. The results represent the protein abundance catalogue of pure classical and enriched non-classical blood peripheral monocytes, and could serve as a reference dataset of the healthy population. The functional analysis of the differences between cell subsets supports the consensus roles assigned to human monocytes.

## 1. Introduction

Monocytes are bone marrow-derived leukocytes with functional capacities including phagocytose, antigen presentation and cytokine production [1]. It was in the late 1980s that Passlik et al. identified different monocyte subsets based on the expression of the surface antigen CD16 [2]. Classical monocytes are critical components of innate immunity, represent the largest population of monocytes, lack CD16 antigen expression (CD14^++^ CD16^−^), and are important scavenger cells [2,3]. Although there are contradictory results, it appears that non-classical monocytes produce more pro-inflammatory cytokines, including TNFα [4,5,6]. Non-classical monocytes appear to be mobilized in different disease scenarios [7,8], and have been considered as an inflammatory monocyte subset in humans. There is an additional monocyte subset called intermediate, which has been proposed as a transitional population bridging the classical and non-classical subsets [3,9]. The different functional properties of monocyte subsets have been widely studied and correlated with different pathogenic conditions. Hence, expansion of non-classical (CD16^+^) and, more consistently, intermediate populations in a wide array of inflammatory and infectious disorders (reviewed in [10,11]) led to the proposal that the definition of monocyte subset frequency could be considered as a biomarker with prognostic value [12]. However, whether CD16^+^ monocytes play a protective or pathogenic role in different diseases is an issue that is still under discussion, and only a comprehensive phenotypic and functional characterization of the different subsets will allow the delineation of their specific association with either disease elimination or progression.

Genome wide gene expression analyses have been conducted to gain functional insights into classical, intermediate and non-classical monocyte subtypes [6,13,14]. Based on this molecular profiling, it has been proposed that classical monocytes are highly versatile cells that mediate anti-bacterial and inflammatory responses, and whose main function is phagocytosis. The non-classical subset, which is more active in T cell stimulation and proliferation, was distinguished by an up-regulation of cytoskeleton rearrangement genes, supporting a patrolling and infiltration function as well as inflammatory cytokine production [6,14]. The intermediate subset appears to be highly related to non-classical monocytes, and is considered to be a transitional subset between the classical and non-classical populations [3,15]. Intermediate monocytes have a pro-inflammatory function showing an enhanced MHCII processing and presentation activity over the non-classical subset [10,11]. Although the transcriptomic signatures have undoubtedly provided remarkable information to differentiate monocyte subsets, a complete biological and functional definition of their proteome under healthy conditions is needed to define the role of monocyte subsets in disease.

The Human Proteome Project (HPP) is an international initiative promoted by the Human Proteome Organization (HUPO) that was conceived in 2010 and launched over the subsequent two years [16,17]. The mission of the HPP is to systematically map and characterize the known proteins encoded by the approximately 20,000 protein coding genes of the human genome [17,18,19]. With the aim of providing a comprehensive map of human proteins in their biological context, the HPP rests on three technological pillars: the shotgun and targeted mass spectrometry pillar (MS), the affinity/antibodies-based pillar (Ab), and an integrated knowledge-based resource. The overall project is organized according to a chromosome-centric strategy (C-HPP), whereby scientific groups from different nationalities agree to characterize the proteome of a selected chromosome following the guidelines of the international consortium and an open-access policy [19,20]. All 24 chromosomes plus the mitochondrial genome-encoded proteome have already been adopted by as many teams from 21 different countries. Knowledge and technical resources generated within the C-HPP initiative are expected to contribute to progress in the understanding and treatment of diseases by the integration and coordination of specific research initiatives through the Biology and Disease (BD)–HPP initiative [20]. Among the 23 BD initiatives currently active, recent developments in cancer proteomics have been summarized highlighting the impressive contribution that the mass spectrometry tool box is already making to reduce the global burden of this disease [21].

In this study, we provide an in-depth analysis of classical (CD14^high^/CD16^−^) and non-classical (CD14^−/low^/CD16^high^) monocyte subsets from 12 healthy individuals. The robustness of the analysis is highlighted by the correlation of data resulting from the analyses performed in five independent laboratories. The protein abundance data provide a comprehensive molecular description of both monocyte populations that supports their functional characteristics. The findings reported here might provide valuable information to further understand the inflammatory process, to make progress in those BD-HPP initiatives focused on topics associated to inflammation, and also accounts for the identification of missing proteins, one of the challenges faced by the C-HPP.

## 2. Materials and Methods

### 2.1. Subjects and Samples

Fresh Anticoagulant Citrose Dextrate Solution A (ACD)-anticoagulated peripheral blood (PB) samples from 12 adult healthy volunteers (10 men and 2 women; median age of 45 years), were used for multiparameter flow cytometry isolation of both classical CD14^high^/CD16^−^ and non-classical CD16^high^/CD14^−/low^ monocyte subsets. Prior to entering the study, patients gave their written informed consent to participate according to the Declaration of Helsinki; the study protocol was approved by the External Ethical Committee of the Spanish National DNA Bank Carlos III (BNADN, University of Salamanca, Salamanca, Spain).

### 2.2. Purification of PB Monocyte Subsets

Isolation of both classical and non-classical PB monocyte subsets was performed within the first 24 h after sample collection using a 4-way fluorescence-activated cell-sorter (FACSAria III, Becton/Dickinson Biosciences –BDB–, San Jose, CA, USA), equipped with the FACSDiva software (BDB). Briefly, peripheral blood mononuclear cells (PBMC) were firstly isolated by a density-gradient centrifugation pre-enrichment step (Ficoll-Paque Plus, GE Healthcare Bio-Sciences AB, Uppsala, Sweden). The remaining erythrocytes were removed from the PBMC by an additional ammonium chloride-mediated red cell lysis step. Prior to sorting, cells were stained with a single 8-color combination of monoclonal antibodies—CD3/CD14/CD16/CD19/CD33/CD45/CD56/HLA-DR—(for details, Table 1), using a direct immunofluorescence flow cytometry technique according to well-established procedures. For PB monocyte subset isolation purposes, classical monocytes were identified as CD14^high^/CD16^−^/CD33^high^/HLA-DR^+^/CD45^high^/CD3^−^/CD19^−^/CD56^−^ cells, whereas non-classical PB monocytes were identified as CD14^−/low^/CD16^high^/CD33^+/low^/HLA-DR^high^/CD45^high^/CD3^−^/CD19^−^/CD56^−^ cells [22]. The purity of each of the isolated PB monocyte population was analyzed with the Infinicyt™ software (Cytognos SL, Salamanca, Spain) and was >98% for classical monocytes. Non-classical monocytes had an average contamination of 30% with intermediate monocytes.

### 2.3. Cell Lysis

Cell pellets were resuspended in lysis buffer (4% CHAPS (*w*/*v*), 7 M urea, and 2 M thiourea) and disrupted by sonication. Cell debris were removed by centrifugation at 12,000 *g* at 4 °C for 10 min. The supernatants were removed and their protein concentration determined by Bradford assay [23]. Samples were frozen and distributed to the different laboratories.

### 2.4. Sample Processing and Mass Spectrometry Analysis

The general procedure was as follows, 20 µg of protein of each sample were fractionated in a 12% polyacrylamide SDS/PAGE. After gel staining with coomassie blue, the lanes were sliced into 10 pieces and digested following standard procedures [24]. The digestion mixture was dried in a vacuum centrifuge and resuspended in 0.1% trifluoroacetic acid. Chromatographic separation was achieved by loading tryptic peptides onto a trap column, desalted, and transferred afterwards onto an analytical column equilibrated in 2% acetonitrile 0.1% formic acid (FA). Elution was carried out with a linear gradient of 2% to 40% B (0.1% FA in acetonitrile) in 120 min at a flow rate of 300 nL/min. Mass spectrometry analysis was performed with different instruments and settings (5600 TripleTOF (SCIEX, Concord, Canada), Impact QTOF (Bruker Daltonics, Bremen, Germany), and LTQ Orbitrap Velos or Q-Exactive (Thermo, Bremen, Germany), depending on the laboratory that processed the sample. The procedure performed in each laboratory may have some minor differences from the general workflow indicated above (Supplementary Table S1).

### 2.5. Shotgun Data Analysis

The datasets were analyzed following the HUPO Guidelines for the identification of proteins using MS/MS experiments. We searched all the mgf files obtained against the neXtProt database (release 20160111) [25] using the target-decoy strategy with an in-house Mascot Server v. 2.3 (Matrix Science, London, UK) search engine. Decoy database was created using the peptide pseudo-reversed method, and separate searches were performed for target and decoy databases. Search parameters were set as follows: carbamidomethyl cysteine as a fixed modification and oxidized methionine as variable modification. Precursor and fragment mass tolerance were set to 20 ppm and 0.5 Da, respectively, and 2 missed cleavages were allowed. False Discovery Rates at PSM level and protein level using Mayu [26] were calculated, and protein identifications were obtained by applying the criteria of PSM FDR < 1% and protein FDR < 1%. Protein inference was performed using the PAnalyzer algorithm [27]. Those proteins not labelled as non-conclusive by this algorithm were considered to be observed proteins in the sample.

The quantification of proteins was performed using a spectral counting approach, the normalized spectral abundance factor (NSAF) [28]. After quality assessment, a filtering process was carried out to eliminate proteins that were not identified in 7 or more samples of a cell type. To normalize each dataset, the logarithmic transformation of the NSAF were corrected by the median so that the distribution was centered around zero. In order to compare with published datasets, the NSAF values were transformed to ppm (parts per million, Table S2) as indicated by Weiss et al. [29]. Hereditary, environmental, and other factors contribute to protein abundance variability [30]. Particularly in human samples, the contribution of these factors to protein abundance is frequently larger than the biological effect under study. To overcome patient variability, we calculated the log2 CD14/CD16 ratios of the protein abundances for each patient. The median of the ratios of each patient was set to zero with the assumption that most protein abundances are essentially the same in these similar cell types [3]. This approach, however, reduces the number of data points and proteins analyzed, since each protein has to be present in both cell types of the same patient to compute the ratio, which is not always the case. To increase the number of proteins in the analysis, we performed an additional significance test comparing the average protein abundance in classical and non-classical monocytes. Proteins were selected as significant using a *p*-value < 0.05 criteria in any of the two approaches using a t-test. In the first method, the significant test was applied only to proteins containing 5 or more patient ratios.

### 2.6. Functional Analysis

Functional enrichment analysis of Gene Ontology (GO) categories was carried out using DAVID [31] and STRING [32]. In the case of DAVID, the whole set of quantified proteins was used as background.

## 3. Results and Discussion

### 3.1. Experimental Design

**Figure 1:**
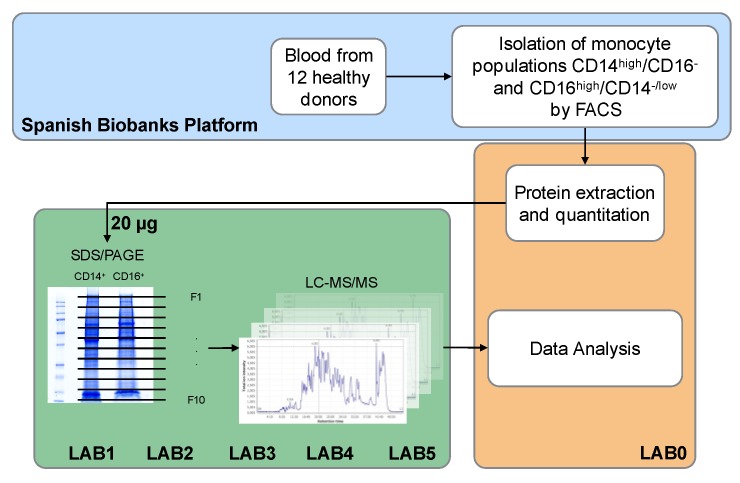
Scheme of the experimental strategy.

**Figure 2:**
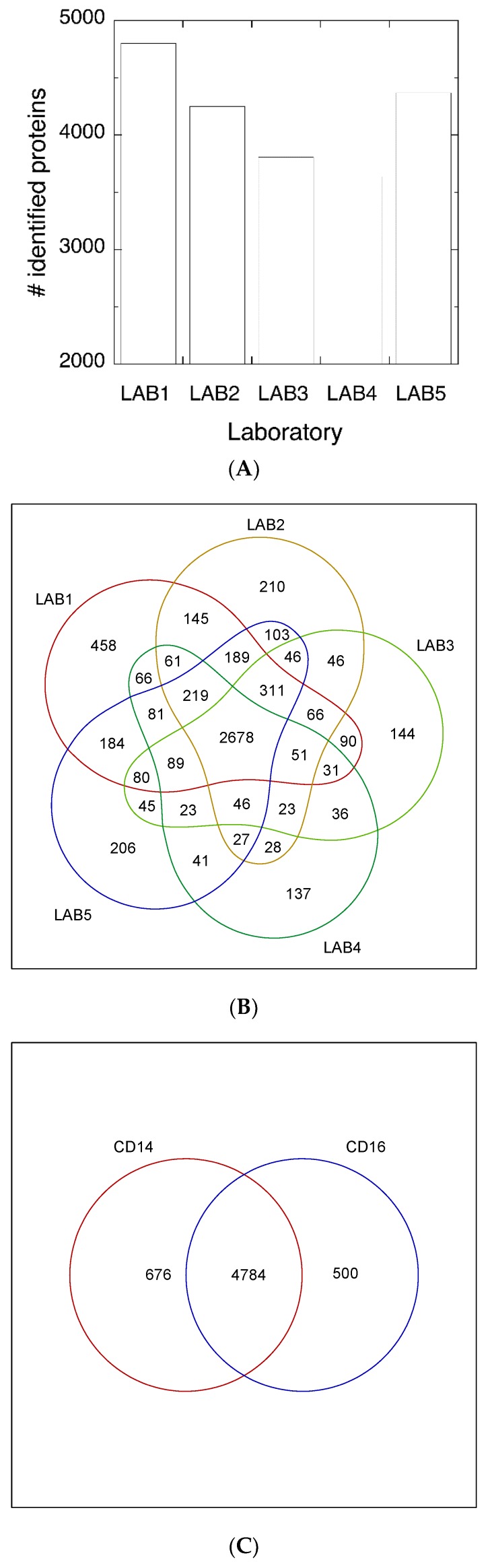

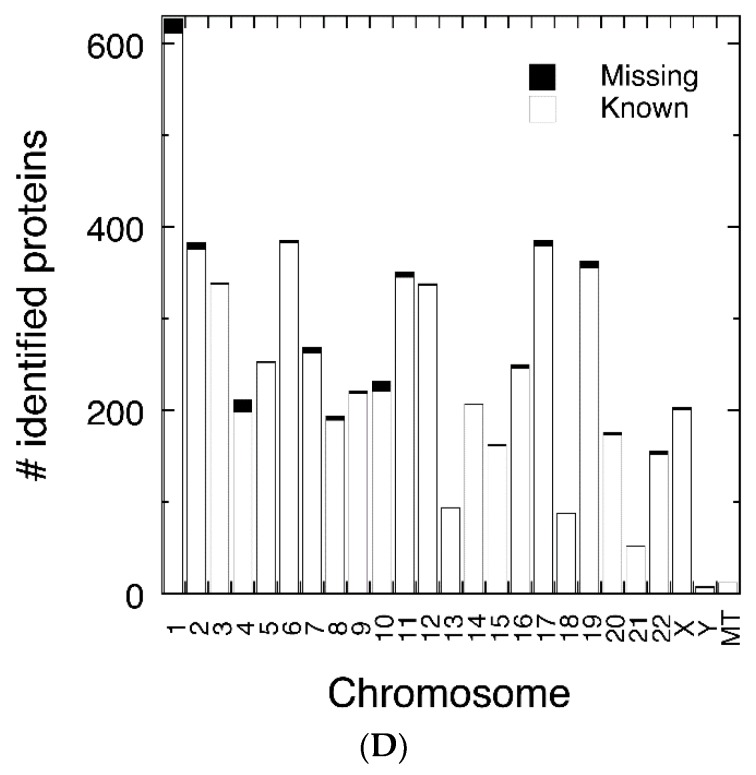
(**A**) Number of total proteins identified in each participating laboratory; (**B**) Venn diagram showing the overlap of the identified proteins among laboratories; (**C**) Venn diagram of identified proteins grouped by cell type; (**D**) Number of identified proteins grouped by chromosome. Missing proteins, according to NeXtprot database criteria, are indicated in black.

The study of monocyte subsets is a very active research field because of their implication in many diseases. The molecular description of these cells should provide important information on their biology and function. There has been some genome-wide transcriptomics analysis [6,13,14] of individual monocyte subsets, but less information at the protein level is available. Most proteomic analyses performed on monocytes have used monocyte derived cell lines or with the whole monocyte population [33,34,35,36,37,38,39,40,41,42]. Wong’s group have carried out proteomic analysis on purified monocyte populations [43,44], where they used iTRAQ to determine differences between cell populations. They used purified classical monocytes, but they did not purify intermediate from non-classical subsets. This mixed population is termed CD16^+^. In this scenario, and under the umbrella of the HPP, we aimed to establish a robust procedure that could be used in any proteomics laboratory to analyze monocyte subsets. Thus, a multicentric proteomic analysis of classical (CD14^high^/CD16^−^) and non-classical (CD14^−/low^/CD16^high^) monocyte subsets derived from 12 healthy volunteer donors was performed in five different laboratories. Our workflow consisted of a protein fractionation by SDS/PAGE followed by the LC-MS/MS shotgun analysis of 10 gel slices (Figure 1). The procedure provides a good compromise between proteome coverage and throughput that could be considered to be a useful proteomic tool for studying and comparing the proteome profile of monocyte subsets under different physiological and pathological conditions. Inter-laboratory experiments are very useful, because they allow the evaluation of the robustness of proteomic workflows. In this context, our results indicate that, despite the number of individuals and laboratories participating in the study, the procedure is quite reproducible. The number of identified proteins ranged from about 3600 to 4800 proteins in the five participating laboratories (Figure 2A), with more than 2600 of them found in all laboratories (Figure 2B). Virtually the same number of proteins were identified in each cell type with more that 85% overlap (Figure 2C). The identified proteins are evenly distributed among chromosomes with an average chromosome coverage of about 30% (Figure 2D), and without any significant difference in chromosome coverage between cell types. Only Y chromosome and mitochondrial DNA, which have a small number of proteins, showed extreme coverage values. According to neXtprot database, 111 of the identified proteins should be classified as missing proteins, since they are within the protein evidence categories 2 and 3 (Figure 2D). However, further investigation will be necessary to confirm these missing proteins.

### 3.2. Protein Abundance

**Figure 3:**
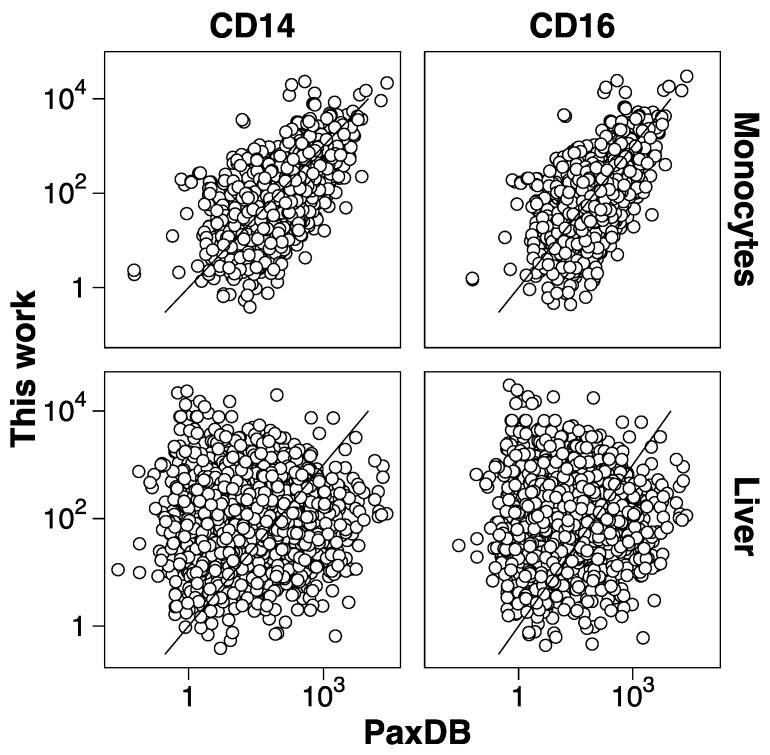
Protein abundance correlation between purified monocyte populations and public datasets obtained from PaxDB. Protein abundances determined in the present work (y-axis) are plotted against public datasets (x-axis). CD14 (classical) and CD16 (non-classical), are shown in left and right panels, respectively. Complete monocyte population and liver datasets are shown in the top panels and bottom panels, respectively.

Protein abundance was estimated by a spectral counting approach; the normalized spectral abundance factor (NSAF). Quantitative methods based in spectral counting are less accurate than other alternatives, such as intensity-based alternatives. However, its simplicity, even with fractionated samples, makes it a very convenient quantitative tool for routinely providing quantitative data. Even in biomarker discovery experiments in a clinical context, where comparison of many samples was necessary, spectral counting quantitation is a valid option [45]. Our NSAF data produced consistent data distribution along samples and laboratories (Figures S1 and S2). After filtering out the low-expression proteins, almost 2600 could be quantitated, and almost all of them (93%) in all five laboratories. The results showed a good correlation among all datasets (Table S4), with a median Pearson correlation coefficient of 0.80 (ranging from 0.66 to 0.93). As expected, the correlation within laboratories was slightly higher (average correlation 0.85) than between laboratories (average correlation 0.79). This degree of reproducibility between laboratories is comparable with results obtained in other multi-laboratory experiments using a more accurate quantitative approach such as SRM [46], indicating the robustness of the experimental procedure. We have estimated that, from 20 µg of starting protein amount, our limit of detection and quantitation is about 20 and 120 pg of protein, respectively. Since previous studies with purified cells used a relative quantitative approach [43,44], this is the first partial protein abundance catalogue of purified classical and non-classical monocyte subsets. There is a good correlation between the protein abundances of classical and non-classical monocyte with the dataset of the complete monocyte population [33] obtained from PaxDb [47] (Figure 3). On the contrary, there is no correlation with a liver dataset, underscoring the significance of the NSAF quantitative data. The observed reproducibility suggests that these datasets could be used as a healthy monocyte reference for comparison with monocytes obtained from different physiological or pathological conditions.

About 100 proteins were differentially expressed—nearly half of them overexpressed—in each cell type. This number is lower than what was observed in an iTRAQ analysis [44]. Although the purities of the CD16^+^ populations used are different, the main difference probably resides in the larger variability of our experimental setup. The inter-individual and inter-laboratory variability introduced in our experimental design certainly increases the magnitude of the biological change required to be detected. Indeed, a considerable inter-individual variability was also observed in a transcriptomic analysis of pure monocyte subsets [6]. In addition, spectral counting quantitative accuracy is lower than iTRAQ. Our experiment was intended to reproduce the working conditions expected to operate in collaborative projects such as the HPP. Thus, the fact that differentially expressed proteins could be detected under these experimental conditions underscores the usefulness of these type of experiments.

### 3.3. Functional Analysis

**Figure 4:**
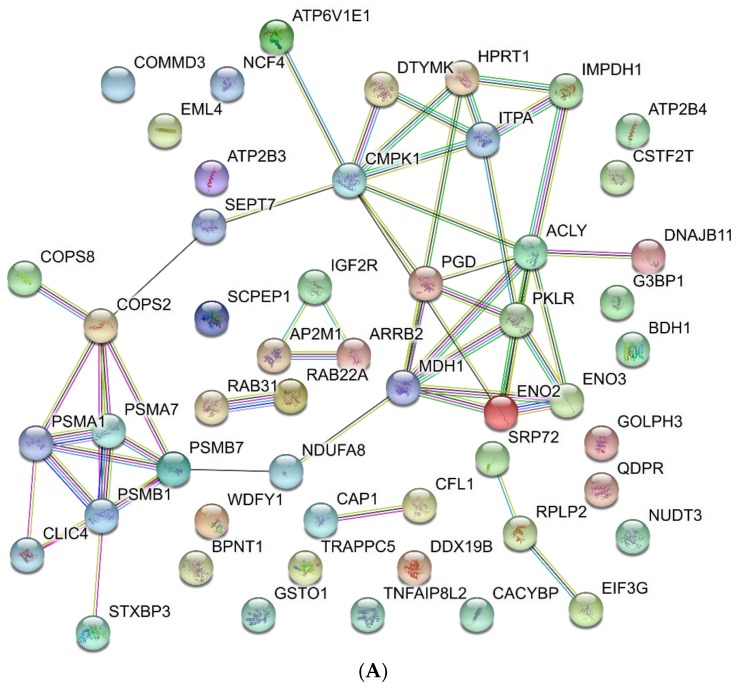

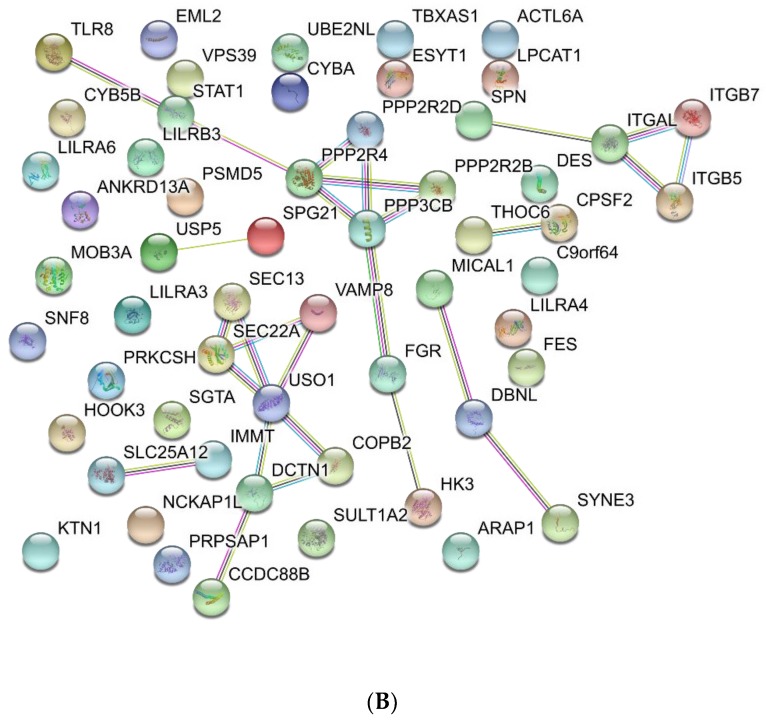
STRING analysis. (**A**) Increased abundance proteins in classical monocytes; (**B**) Increased abundance proteins in non-classical monocytes. STRING color code for interacting lines: cyan: curated databases; magenta: experimentally determined; green: gene neighborhood; red: gene fusions; blue: gene co-occurrence; golden: textmining; black: co-expression; purple: protein homology.

The functional analysis of the differentially expressed proteins indicates that they are biologically related (Figure 4). The biological processes in which differential proteins are involved support the consensus functions assigned to these cell types [5] and reviewed in [10,11]. Non-classical monocytes have been proposed to have an important role in anti-viral immunity, which is mediated by TLR 7/8 receptors. They have also been assigned a patrolling function, and proposed to induce T cell proliferation and activation. Indeed, the abundance of some of the proteins involved in these processed were observed to increase in the non-classical subset (Figure 4B). In accordance with these functions, some of the GO terms enriched in these cells are related to the immune system response (Supplementary Tables S6 and S7). It is interesting to note that integral membrane proteins are also enriched (Supplementary Tables S6 and S7). This result is, at least in part, due to the fact that most of the identified protein related to immune system responses are membrane proteins. Frequently, membrane proteins are underrepresented in proteomic experiments; probably because of their low solubility. Thus, the enrichment of membrane proteins in this fraction is an unexpected result. The use of an SDS/PAGE step to fractionate proteins may have contributed to overcome the low solubility of membrane proteins and allow their enrichment. This is particularly useful for studying the biology of monocyte populations, since membrane proteins are the main determinants for their classification, purification and manipulation.

The main function of the classical subset is phagocytosis. Phagocytosis and ROS production, both of which are reduced in non-classical monocytes [5], are associated with a metabolic reprogramming. In macrophages M1, which are derived from classical monocytes, a switch from oxidative phosphorylation to glycolysis has been described [48]. This is like the Warburg effect observed in proliferating tumor cells, although, in this case, the high glycolytic flux is not required for proliferation, but for ROS production. The increase in glycolysis also produces increases in the pentose phosphate pathway and nucleotide metabolism, which are needed for the production of NADPH used to produce ROS. Supporting this metabolic switch, among the proteins with increased abundance, those involved in nucleotide and glucose metabolism are significantly enriched (Figure 4A, and Supplementary Tables S6 and S7).

## 4. Conclusions

We have performed a multicentric proteomic characterization of the classical and non-classical monocyte populations using a relatively simple experimental approach. The high reproducibility of the results obtained in different laboratories demonstrated the robustness of the procedure. The obtained results constitute the first protein abundance catalogue of pure classical and non-classical monocyte populations and its functional analysis supports the established functions of these cell types. The results can be a useful proteomic tool for the study of the biology of monocytes in different pathological conditions serving as a reference set of healthy individuals.

## Figures and Tables

**Table 1:** Immunophenotypic markers used for the identification and isolation of both classical CD14^high^/CD16^−^ and non-classical CD16^high^/CD14^−/low^ monocyte subsets.

Marker	Fluorochrome	Clone	Source
CD3	APC-H7	SK7	BD Bioscience ^1^
CD14	FITC	47-3D6	Immunostep ^2^
CD16	PE-Cy7	3G8	BD Bioscience ^1^
CD19	APC	A3B1	Immunostep ^2^
CD33	PerCP-Cy5.5	P67.6	BD Bioscience ^1^
CD45	PO	HI30	Immunostep ^2^
CD56	PE	C5.9	Cytognos ^3^
HLA-DR	PB	L243	Biolegend ^4^

APC-H7, allophycocyanin hilite 7; FITC, fluorescein isothiocyanate; PE-Cy7, phycoerythrin cyanin 7; APC, allophycocyanin; peridinin chlorophyll protein–cyanin5.5; PO, pacific orange; PE, phycoerythrin; PB, pacific blue. ^1^ BD Biosciences, San Diego, CA, USA; ^2^ Immunostep SL, Salamanca, Spain; ^3^ Cytognos SL, Salamanca, Spain; ^4^ Biolegend, San Diego, CA, USA.

## Supplementary Materials

The following are available online at http://www.mdpi.com/2227-7382/6/1/8/s1, Figure S1: Box Plot of the log(NSAF) of identified proteins, Figure S2: Box Plot of the log(NSAF/Med) of quantified proteins, Table S1: Details of the sample processing and mass spectrometry used in each laboratory, Table S2: Identified proteins, Table S3: Quantified proteins, Table S4: Correlation analysis of all quantitation datasets, Table S5: Differential proteins, Table S6: Functional enrichment results from STRING, and Table S7: Functional enrichment results from DAVID.
