# Supplementary figures and images for: In-Depth Proteomic Characterization of Classical and Non-Classical Monocyte Subsets
Source: Proteomes. 2018 Feb 5;6(1):8. doi: 10.3390/proteomes6010008 (PMC5874767; doi:10.3390/proteomes6010008)

$\log(\text{NSAF})$

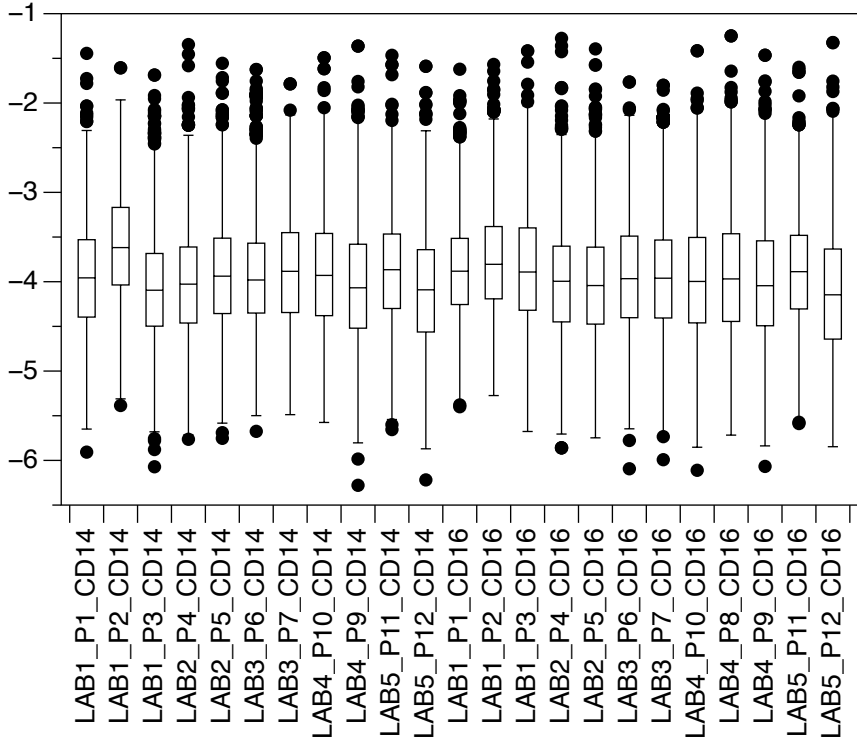

Supplement: Supplementary file 1 [file proteomes-06-00008-s001.zip › Figure S1.pdf]

$\log(\text{NSAF}/\text{Med})$

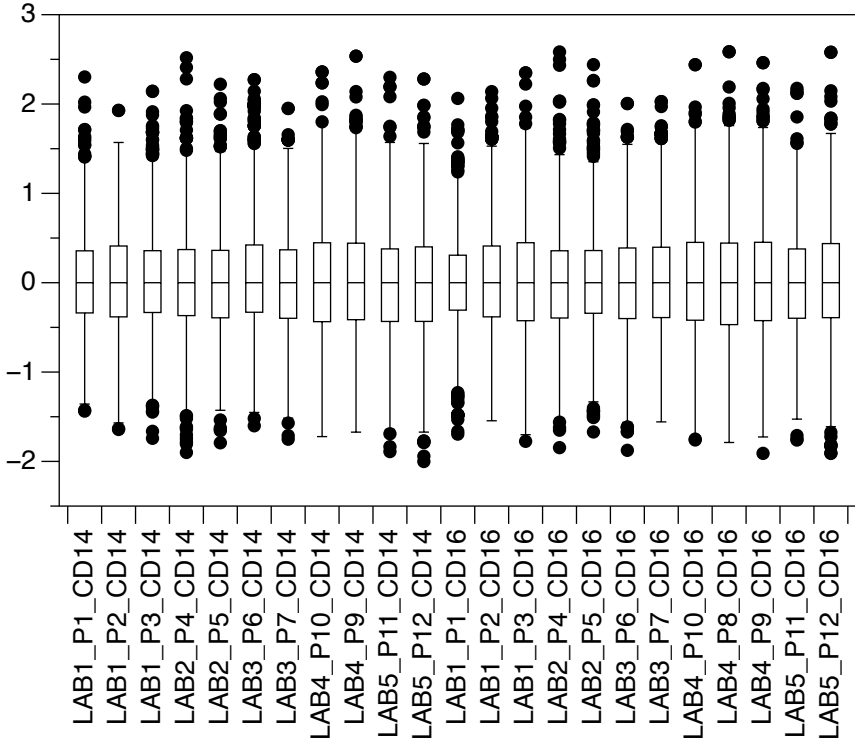

Supplement: Supplementary file 1 [file proteomes-06-00008-s001.zip › Figure S2.pdf]
